# Hepatic perivascular epithelioid cell tumor (PEComa): contrast-enhanced ultrasound (CEUS) characteristics—a case report and literature review

**DOI:** 10.1007/s12328-023-01779-w

**Published:** 2023-03-25

**Authors:** Sami Matrood, Christian Görg, Ehsan Safai Zadeh, Amjad Alhyari

**Affiliations:** 1 Department of Gastroenterology, Endocrinology, Metabolism and Clinical Infectiology, University Hospital Marburg, Philipps-University Marburg, Marburg, Germany; 2 Interdisciplinary Centre of Ultrasound Diagnostics, University Hospital Marburg, Philipps-University Marburg, University Hospital Giessen and Marburg, Baldingerstraße, 35042 Marburg, Germany

**Keywords:** PEComa, Liver tumors, Ultrasound, CEUS

## Abstract

Perivascular epithelioid cell tumors (PEComa) are rare mesenchymal neoplasms that arise from soft tissue of various organs such as the stomach, intestines, and lungs. We report a rare case of a primary PEComa of the liver and its characteristics on contrast-enhanced ultrasound (CEUS) in a 51-year-old female patient with an incidental finding of a hypoechoic liver lesion with peripheral hypervascularization on Doppler ultrasound. CEUS showed homogenous hypervascularity in the arterial phase that was consistent in the portal phase. In the late phase, a central washout phenomenon was evident. Histopathologic findings on sonographic biopsy of the lesion revealed a mesenchymal tumor with positivity for melanocytic markers Human Melanin Black-45 (HMB45) and Melan-A consistent with a PEComa. Despite the absence of high-risk features for malignancy, surgical resection was recommended due to the uncertain malignant potential of PEComas. The patient refused the operation and preferred sonographic follow-up; the lesion was stable over a period of 2 years. CEUS can provide valuable information regarding PEComa. After histological confirmation, the choice between resection and a watchful waiting must be made on individual basis.

## Introduction

PEComas are rare mesenchymal tumors that histologically present as a composite of epithelioid or spindle-shaped mesenchymal cells that immunohistochemically show a characteristic concomitant expression of melanocytic Human Melanin Black-45 (HMB45) and Melan-A and smooth muscle markers (actin and desmin) [1, 2].

For the most part, PEComas affect women with predilection for the uterus [2]. However, PEComas can also occur in other organs such as the stomach, intestine, and lungs [3]. Hepatic PEComas have been described in only a few dozen cases [2, 4]. Hepatic PEComas are often discovered incidentally because of their mostly asymptomatic course [2, 4].

Contrast-enhanced ultrasound (CEUS) is of great importance for further differentiation of incidentally detected focal liver lesions [5]. However, the peculiarities of PEComas on CEUS are described in only five reports, whereby CEUS cannot provide certainty in diagnosis and histologic confirmation remains essential [4, 6–9]. The present case report describes the CEUS pattern of another histologically confirmed PEComa.

## Case report

**Fig. 1:**
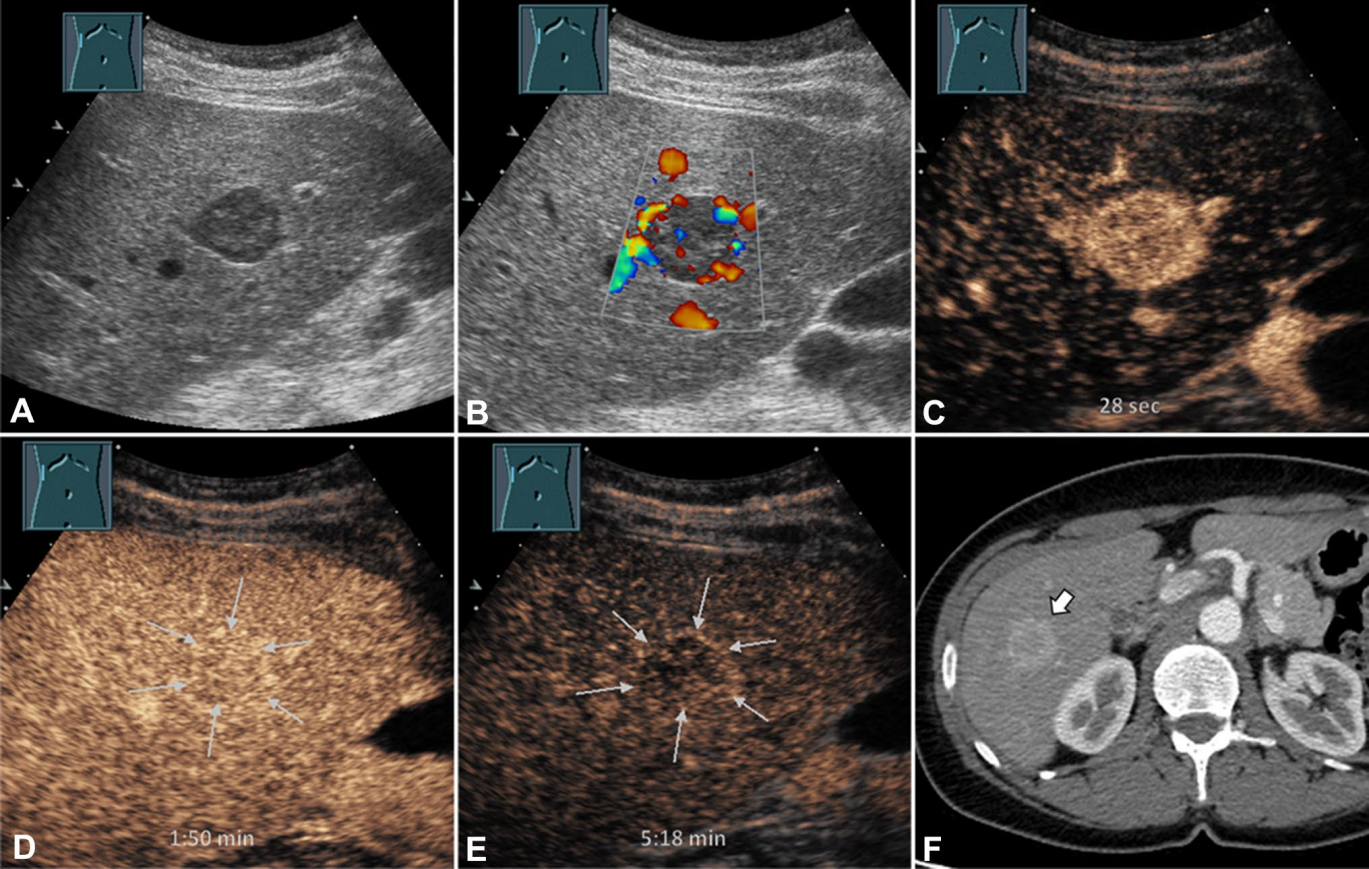
(**A**) B-mode ultrasound of a 51-year-old asymptomatic patient with hepatic PEComa shows a hypoechoic lesion in segment V of an otherwise unremarkable liver. (**B**) On color Doppler ultrasound peripheral flow signals were seen. Contrast-enhanced ultrasound of the liver in a patient with proven PEComa (**C**–**E**). The lesion shows a marked homogeneous enhancement 28 s after administration of SonoVue® (**C**). In the portal phase, an isoenhancement of the lesion (arrows) persists (**D**). In the late phase, mild central washout with a persistent rim enhancement of the lesion (arrows) is recognizable (**E**). Computed tomography (courtesy of Professor Dr Mahnken, Department of Radiology, University Hospital Marburg) shows a hepatic PEComa with strongly accentuated rim in the arterial phase (**F**)

A 51-year-old Caucasian woman of normal weight (167 cm, 68 kg) with refractory arterial hypertension underwent a sonographic examination to exclude renal artery stenosis at our ultrasound unit. As an accidental finding, a 2 cm sized focal solid hypoechoic lesion in the right lobe of an otherwise homogenous liver was detected (Fig. 1A). The sonographic findings of the remaining abdominal organs were unremarkable and renal artery stenosis could be excluded.

At the time of presentation, the patient was asymptomatic without any complaints related to her liver lesion. Fever, night sweats, weight loss, or other vegetative symptoms were denied. The patient had no relevant previous medical history that could indicate a particular nature or origin of the liver lesion. The patient’s physical examination and the laboratory workup including liver enzymes and blood count were inconspicuous.

The hypoechoic liver lesion showed a peripheral flow signal on color Doppler sonography (CDS) (Fig. 1B). CEUS with the ultrasound contrast media SonoVue_®_ (Bracco S.p.A., Milan, Italy) was performed for further evaluation. In the arterial phase, a marked hyperenhancement was seen with an isoenhancement in the portal venous phase and a light wash out in the parenchymal phase (Fig. 1C–E). A diagnosis of a hepatocellular adenoma (HCA) was initially suspected, and for further differentiation, an ultrasound-guided biopsy was performed.

**Fig. 2:**
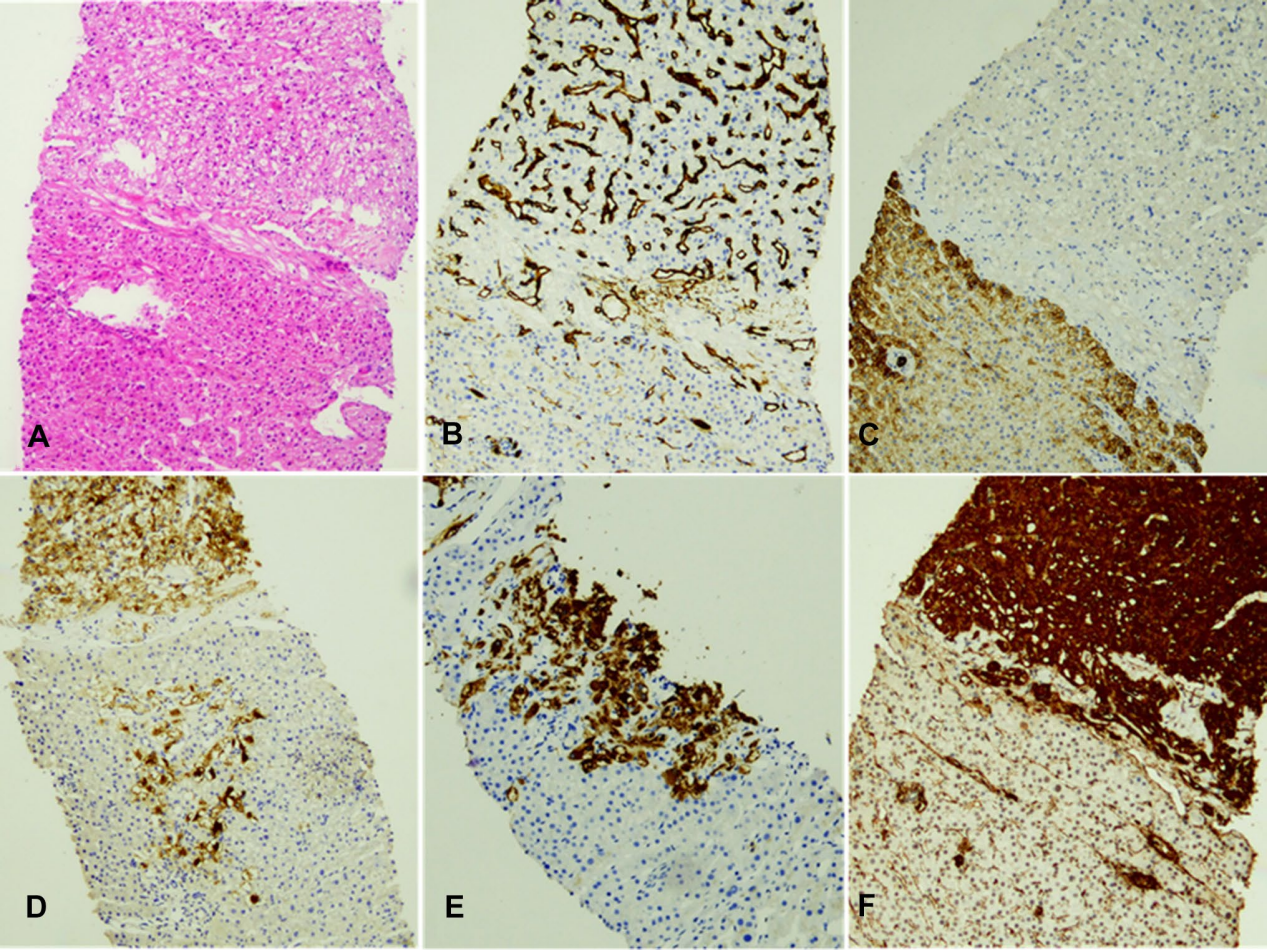
Core needle biopsy of a 2 cm suspicious liver nodule. Hematoxylin–eosin staining (**A**) showing regular liver tissue and a mesenchymal tumor, consisting of spindle-shaped cells with partly clear cytoplasm surrounding numerous, partly oddly shaped blood vessels. Immunohistochemical staining against CD34 (**B**) highlights endothelial cells lining small and medium sized, partly oddly shaped vessels between the tumor cells (upper half). Many tiny, rounded vessels are only discernible with this staining. The tumor cells appear more epithelioid here. CD34 also highlights small vessels within the regular liver tissue (lower half). Immunohistochemical staining against CKMNF116 (**C**) illustrates the mesenchymal nature of the lesion with negative tumor cells (upper half) against regular liver tissue stained positively (lower half). Immunohistochemical staining against HMB45 (**D**), Melan-A (**E**) and smooth muscle actin (**F**) demonstrates myomelanocytic differentiation of the tumor cells. Scale bars, 100 μm, respectively

The histological examination revealed a mesenchymal tumor composed of spindle cells with a marker profile consistent with a PEComa (Fig. 2). Immunohistochemical staining showed positivity for smooth muscle actin, HMB45 and Melan-A in the tumor cells, whereas the adjacent liver tissue was negative for HMB45 and Melan-A (Fig. 2D, E). No high-risk features such as marked nuclear atypia, increased mitotic activity, necrosis, infiltrative growth, or vascular invasion were present, indicating a benign nature of the lesion [4, 10].

On contrast-enhanced abdominal computed tomography (CT), the lesion showed a rim enhancement in the early arterial phase and an isoenhancement in the portal venous phase (Fig. 1F). No further lesions were detected on abdominal CT. The CT of the chest showed a 6 mm-sized nodule in the right lung, which was described to be most consistent with a small granuloma. The patient underwent both upper and lower gastrointestinal endoscopies with normal findings. The interdisciplinary tumor board recommended surgical resection of the PEComa due to the unknown malignant potential [4], and a follow-up of the pulmonary nodule [11].

After being informed about the recommendation for resection, the patient opted for a sonographic follow-up of her liver PEComa. The lesion was closely monitored on ultrasound initially every three months. The pulmonary nodule remained unchanged in chest CT after 6 months, so no malignancy was suspected.

No new lesions were detected and the size, sonographic features on grey-scale, and the behavior of the lesion on CEUS remained unchanged over a period of 2 years of sonographic follow-up.

## Discussion

PEComas are rare mesenchymal neoplasms composed of perivascular epithelioid cells [1]. It represents a family of tumors which includes also angiomyolipoma, lymphangioleimyomatosis, rare clear-cell tumors like clear-cell “sugar” tumor of the lung and clear-cell myomelanocytic tumor of the falciform and round ligament of the liver [18]. The diagnosis of PEComa is dependent on histological confirmation, where immunohistochemical detection of HMB45 and Melan-A is decisive [2]. PEComas are usually sporadic, although in rare cases, PEComas are related to alterations in tuberous sclerosis complex [3, 12]. The histopathological differential diagnosis of liver PEComa includes hepatic adenoma, malignant melanoma, gastrointestinal stromal tumor (GIST), gastrointestinal neuroectodermal tumor, metastatic renal cell carcinoma, and metastatic adrenal cortical carcinoma. In addition, PEComas without adipocytic differentiation may mimic hepatocellular carcinoma (clear-cell variant) and cholangiocarcinoma requiring immunohistochemical evaluation for respective tumor markers [19].

A literature review by Liu et al. on a total of 20 cases of hepatic PEComas showed that with 18 out of 20 cases, PEComas mostly affect women and that 9 out of 20 patients were asymptomatic incidental findings [13]. The symptomatic patients complained of abdominal pain or abdominal discomfort [13]. All of this PEComas were shown to be benign lesions.

The patient presented showed no abnormalities in the laboratory tests consistent with other reports of PEComas [2, 14]. Due to the lack of a clinical presentation, the differentiation of PEComas from other incidentally found solid liver lesions is first based on imaging. Ultrasonography and especially CEUS play a predominant role in the diagnosis of incidentally found solid liver lesions, with regard to the question of whether a histologically guided biopsy is indicated [5, 15].

Consistent with previous reports, on ultrasound the PEComa appeared as a well-demarcated hypoechoic lesion, while peripheral hyperperfusion was evident on CDS [4, 6–8]. Early arterial hyperenhancement of the PEComa with preserved isoenhancement in the portal phase and a light parenchymal washout phenomenon were consistent with features in CEUS of previously reported hepatic PEComas [4, 6–9] (Table 1).

**Table 1:** Case reports of hepatic PEComas with characterization by ultrasound and CEUS

ID	Reference	Sex/age at diagnosis	Ultrasound	Color doppler ultrasound	Contrast-enhanced ultrasound
I	Della Vigna P. et al. [8]	f/46	Well-demarked hyperechoic tumor with a diameter of 35 mm in S3	Peripheral hyperperfusion	Arterial phase: homogenous hyperenhancement Portal phase: homogenous isoenhancement Late phase: weak protracted hyperenhancement
II	Akitake R. et al. [7]	f/36	Well-demarked isoechoic tumor with a diameter of 35 mm in S2	Vessels encircled the tumor	Hyperenhancement in arterial phase with fast washout within few seconds suggesting an arteriovenous shunt and hypervascularity
III	Panahova et al. [4]	m/38	Inhomogeneous tumor with hypoechoic rim	Peripheral hyperperfusion	Arterial phase: hyperenhancement Portal phase: isoenhancement Late phase: inhomogeneous central washout
IV	Dezman R. [8]	f/24	Well-demarked, hypoechogenic tumor, 20 mm in diameter	Peripheral hyperperfusion	Arterial phase: homogenous hyperenhancement Portal phase: iso- to hyperenhancement Late phase: iso- to hyperenhancement
V	Li C. [9]	f/36	Heterogeneous hypoechoic tumor, 30-40 mm in diameter	Peripheral hyperperfusion	Arterial phase: hyperenhancement Portal phase: isoenhancement Late phase: hypoenhancement
VI	Present case (2022)	f/51	Well-demarked, hypoechoic tumor with a diameter of 20 mm	Peripheral hyperperfusion	Arterial phase: hyperenhancement Portal phase: isoenhancement Late phase: central washout

Due to the few cases of PEComas described so far, diagnosis by CEUS based on these characteristics is not sufficient. Furthermore, according to World Federation for Ultrasound in Medicine and Biology (WFUMB) guidelines on hepatic CEUS, a hepatic adenoma, a focal nodular hyperplasia or a well-differentiated hepatocellular carcinoma should be considered differential diagnoses to this CEUS pattern [15].

In the literature, contrast enhancement of PEComas on CT scans shows heterogenous hyperenhancement in the arterial phase and a decrease in enhancement in the portal phase [4]. In the present case, the hepatic PEComa was characterized by a rim enhancement in the early arterial phase (Fig. 2A–C) as described in Panahova et al. and Krawczyk et al. [2, 4]. According to the patient’s desire of a conservative management a wait-and-watch strategy using regular CEUS examinations was undertaken. In one report, the MRI appearance of a benign PEComa was reported to be hypervascular with hypointense signal on T1 and hyperintense signal on T2 and no increased tracer accumulation on PET-CT [18]. In fact, PET-CT may be helpful in providing additional information regarding the malignant nature of PEComas since it was reported that malignant PEComas demonstrate higher tracer uptake and most benign PEComa have exhibited no increased tracer uptake [20].

Histologically, most hepatic PEComas appeared benign, while only few cases of malignant hepatic PEComas were reported [4, 16, 17]. However, due to the unknown risk of malignant transformation, resection should still be considered, especially as there are no established therapies for advanced stages of PEComas. The case description by Panahova et al. shows that a punch biopsy alone may not be sufficient for the assessment of malignancy, as only the surgical resectate revealed invasive growth and an increased mitotic rate [4].

A retrospective single-center study by Chen et al. suggested the use of a watch-and-wait strategy for PEComas with benign pattern, although this study only included surgically resected or locally ablated patients [14]. Overall, there is a lack of long-term experience on the treatment of hepatic PEComas, especially when considering a wait-and-watch strategy.

## Conclusion

Hepatic PEComa is a rare form of hepatic neoplasms with still uncertain malignant potential. Due to the few cases described so far, a reliable diagnosis by CEUS is not possible, so that histological confirmation should also be considered. Surgical resection still seems to be indicated as the primary therapy for localized PEComas. When using a watch-and-wait strategy in benign PEComas, close monitoring should be carried out due to the unknown potential for malignant transformation.
